# Evolution is driven by natural autoencoding: reframing species, interaction codes, cooperation and sexual reproduction

**DOI:** 10.1098/rspb.2022.2409

**Published:** 2023-03-08

**Authors:** Irun R. Cohen, Assaf Marron

**Affiliations:** ^1^ Department of Immunology and Regenerative Biology, Weizmann Institute of Science, Rehovot 76100, Israel; ^2^ Department of Computer Science and Applied Mathematics, Weizmann Institute of Science, Rehovot 76100, Israel

**Keywords:** interaction, autoencoding, species interaction code, sexual reproduction, survival of the fitted

## Abstract

The continuity of life and its evolution, we proposed, emerge from an interactive group process manifested in networks of interaction. We term this process *survival of the fitted*. Here, we reason that survival of the fitted results from a natural computational process we term *natural autoencoding*. Natural autoencoding works by retaining repeating biological interactions while non-repeatable interactions disappear. (i) We define a species by its *species interaction code*, which consists of a compact description of the repeating interactions of species organisms with their external and internal environments. Species interaction codes are descriptions recorded in the biological infrastructure that enables repeating interactions. Encoding and decoding are interwoven. (ii) Evolution proceeds by natural autoencoding of sustained changes in species interaction codes. DNA is only one element in natural autoencoding. (iii) Natural autoencoding accounts for the paradox of genome randomization in sexual reproduction—recombined genomes are analogous to the diversified inputs required for artificial autoencoding. The increase in entropy generated by genome randomization compensates for the decrease in entropy generated by organized life. (iv) Natural autoencoding and artificial autoencoding algorithms manifest defined similarities and differences. Recognition of the importance of fittedness could well serve the future of a humanly livable biosphere.

## 1. Background and aims

Previously, we proposed:

(1) that interactions are the vehicle of biological evolution—what evolve are the interactions of entities;
(2) that cooperative group interaction networks are more functional in evolution than are individual competition and survival of the fittest—the outcome of evolution is not survival of only the reproductively dominant individuals but survival of integrated group networks—we have termed this outcome *survival of the fitted* [1–3]; and
(3) that evolution takes place in accord with the laws of physical nature, including the dissolution of order dictated by the second law of thermodynamics and the continuous increase of entropy [3].

**Figure 1.**
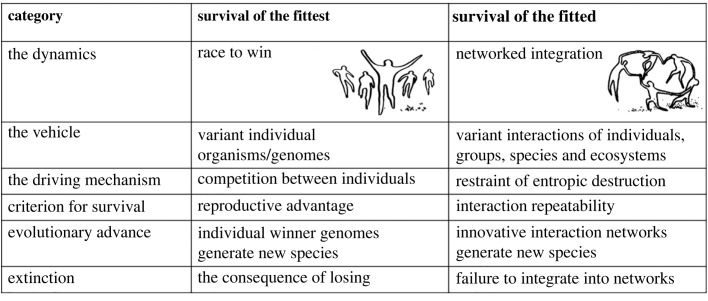
Two mechanisms of evolution: differences between *survival of the fittest* and *survival of the fitted*. Image credits: Henri Matisse; Rachel Shiloach.

Figure 1 summarizes salient differences between survival of the fittest and survival of the fitted.

This paper extends these ideas and supports two conclusions: first, that species can be defined as descriptions of codes of interaction common to the collective of species organisms; and second, that a computational enterprise we term *natural autoencoding* generates the evolution of species and of the biosphere. Below, we define some of the key terms we use in developing these ideas.

## 2. Basic definitions

### (i) Evolution

Evolution is the narrative of changes in species and their interactions over time [4].

### (ii) Environment

The environment is the aggregate of the living and non-living entities, structural and dynamic, within which an organism exists and operates.

### (iii) Survival of the fittest; natural selection

Survival of the fittest is a term used since Darwin to describe the mechanism that drives evolution [5]. Survival of the fittest assumes a continuous struggle of variant individual organisms for survival and reproductive advantage in the face of limited resources. This struggle leads to survival of the *fittest* individuals and their dominion over less fit individuals; predominating proliferation of the winners determines the characters of species. This process of reward is termed *natural selection*. Variants of natural selection have been proposed to account for advantages in cooperation as well as in struggle [6].

### (iv) Survival of the fitted

It is now clear that all living systems—cells, organisms, species (including *Homo sapiens*) and ecosystems—survive in extensive networks of interaction and group cooperation [2,3,7]. A few examples include the dependence of every multicellular organism on a resident microbiome [8]; the symbiotic web of forest trees and fungi [9]; and the collaboration and symbiosis that create a coral colony [10]. The biosphere is sustained by such repeatable interactions; the biosphere is a *worldwide web* of interactions.

Survival of the fitted is an alternative mechanism to account for evolution [1–3]. Survival does not result only from individual struggles for reproductive advantage; surviving organisms are those that integrate into networks of sustaining interactions; longevity and rates of reproduction are not individual achievements but to a large extent are expressed in the lifestyle of the species—some species of organisms live and reproduce for a season, some for a century, or more; some produce many offspring and some few.

Survival of the fitted affirms that what works, works [3]. By contrast, Darwinian survival of the fittest would claim that what wins works.

### (v) Interaction

Interactions are mutual relationships between two or more entities (termed interactors) in which the interactors transmit or exchange energy, matter or information; an exchange of information often involves matter and energy. A *process* is an ordered set of interactions.

Sustainable interactions are characterized by *repetition and sequence*. Metabolic interactions, for example, are organized in repeating, sequential pathways—in each pathway one interaction is connected to the next in line [11].

Cycles of reproduction, growth, ageing, illness, predation and death are accessible examples of the universality of repeated, sequential interactions.

### (vi) Information and meaning

We define information according to Shannon as a particular non-random structure or arrangement of entities or processes [12,13]. Arrangements bear information; but an arrangement by itself has no meaning unless it interacts with other arrangements to produce some effect [1,12]. The consequences of the interactions of information constitute the *meaning* of the information. A sequence of DNA, for example, bears information that only gains meaning through expressed interactions including transcription and translation [14]. Written words, too, have no meaning unless somebody or some thing can read them. The meaning of information emerges from the information’s interactions.

### (vii) Energy and matter

Energy, in functional terms, is the impetus behind motion and activity [15], including the capacity to do work. Energy enables interactions.

Matter can be viewed as a product of interaction: the nuclei of atoms are created by interactions between fundamental particles; atoms are formed by interactions between nuclei and electrons; and molecules are formed by interactions between atoms.

So one can conclude that anything made of atoms or molecules, including living entities and the biosphere itself, is made of interactions. As stated by Feynman [16, prologue] and others [17], interactions constitute reality. This view is also in line with the relational philosophy of Leibniz & Whitehead [18], [19, ch. 18].

### (viii) Code, encoding and decoding

The word *code* can be defined in different ways [20]. The word is derived from the Latin *codex*, a book. We here define an *interaction code* as a description (a ‘book’) that outlines steps that, when implemented, are able to convert one form of biological information into another form of biological information. A biological code is analogous to the text of a computer algorithm that describes a set of repeatable interactions.

The term code can be used in at least four interwoven contexts. This is exemplified by the genetic code.

(1) A concrete instance of input for a translation process: One concrete codon UUU is a code for generating molecules of phenylalanine.
(2) A single decoding rule: *All codons UUU are codes for the molecules of amino acid phenylalanine*, or *UUU maps to phenylalanine*.
(3) A description of a set of reactive behaviours: a DNA sequence that translates into a particular protein is a code for all the reactive behaviours of this protein. Actually, the produced protein is another code for these reactions.
(4) A complete system for such rules: this is exemplified by the very concept of the genetic code. Other such systems include the binary code used in computing to encode numbers, or the Dewey Decimal System for encoding book locations by subject in a library. Below we describe natural autoencoding, which is a process that forms code systems in nature.

A code may also feature information that summarizes or reduces to essentials the interactions that gave rise to the encoded information. For example, a DNA code expresses the essence of the myriad of biological interactions—molecular, physiological and evolutionary—that have resulted in that sequence of DNA. All of these many complicated interactions are reduced to the DNA sequence; this reduction encapsulates all the foregoing networks of interactions into a concise, manageable and functional code molecule, one that can be replicated and transmitted.

### (ix) Encoding

The term *encoding* refers to the process by which anterior interactions give rise to a derivative, often simplified new entity—the code. The code, as we defined above, is a description; encoding, unlike the derived code, is a process generated by actual interactions.

### (x) Decoding

*Decoding*, like encoding, also is a process—the interactions by which the encoded potential interactions get expressed—become actualized. Decoding is a process that generates new information through interactions. The distinction between description—code—and process—the encoding/decoding interactions—is useful; in the course of our discussion, we shall relate this distinction to a natural autoencoding mechanism of evolution.

### (xi) Natural autoencoding

Below we shall analyse this concept in detail, but we introduce it here among our definitions. Natural autoencoding is a process by which repeating patterns of encoding and decoding, a complete code system, are formed and maintained.

## 3. Species interaction code

The concept of species, since Darwin, is linked to evolution; this link is reflected in the title of Darwin’s foundational work, *The Origin of Species* [5]. A species originally referred to entities that look alike—the word *species* derives from the Latin *specere*, 'to see'. Living species, basically, are composed of types of creatures that look alike, and interact alike.

The definition of a species beyond appearances is controversial. A search in Google Scholar for *species* returns millions of publications, but there is not one universally accepted definition; researchers have proposed many different definitions of multicellular species based on morphology, genetics, sexual reproduction, ecology and other criteria [21].

The definition of bacterial species is even more uncertain [22] and we shall not deal in depth with prokaryotes or single-cell eukaryotes in this paper. Unless designated otherwise, here the word species refers to multicellulars.

We define a species as a collective of organisms that jointly carry out a set of potential, repetitive interactions with their external and internal environment.

The code of each species is the ‘book’ describing the essential interactions carried out by members of the species.

Note that not every individual organism within a species need perform all the coded interactions of the species—males, females and particular ‘sub-types’ of organisms within a species can perform uniquely different interactions that, nevertheless, are included in the collective species code. This is because the ensuing generations of species organisms, as a group, continue to collectively fulfil the species code, maintained, but not necessarily performed, by the reproducers.

Worker bees, for example, cannot themselves reproduce, but reproduction by queen bees and fertile males will continuously generate worker bees as part of the coded description of bees; likewise, the queen bee will never make honey, but the non-reproducing workers will.

In §8 below, we discuss the function of sexual reproduction in maintaining and defining a multicellular species. Organisms may exist in close connection with other organisms, but the species can be distinguished by independent sexual reproduction. For example, every multicellular organism is accompanied by a resident microbiome, but the microbiome is not a member of the multicellular species; the microbiome organisms are not reproduced by the act of sexual reproduction of the multicellular organism—the microbiome is not part of the organism species, but has to be acquired independently.

The code of species interactions comprises the information that, when decoded and expressed, enables the species to survive and thrive in the context of its environment.

The concepts of interaction code and species interaction code are different. Above in §2, we defined a code for a given interaction, interaction code, as a description of the interaction. A species interaction code, however, has a broader meaning: a species interaction code is a description of *sets* of interactions carried out at different times by the species collective; a species interaction code is an array of interaction codes.

Barbieri has pointed out the importance of codes in living systems generally; he proposed that life emerges from codes that enable the maintenance and the development of structures and processes, including the genetic code and its expression; on this basis, he developed the concept of ‘codepoiesis’, the idea that living systems function to preserve organic codes and to evolve by developing new codes. Barbieri defines a code as ‘a mapping between the objects of two independent worlds’ [23,24]. Species interaction codes, by contrast, are not mappings between ‘independent worlds’; rather they are descriptions of sets of mutually dependent interactions that link organisms to their specific environments and ecosystems.

**Figure 2.**
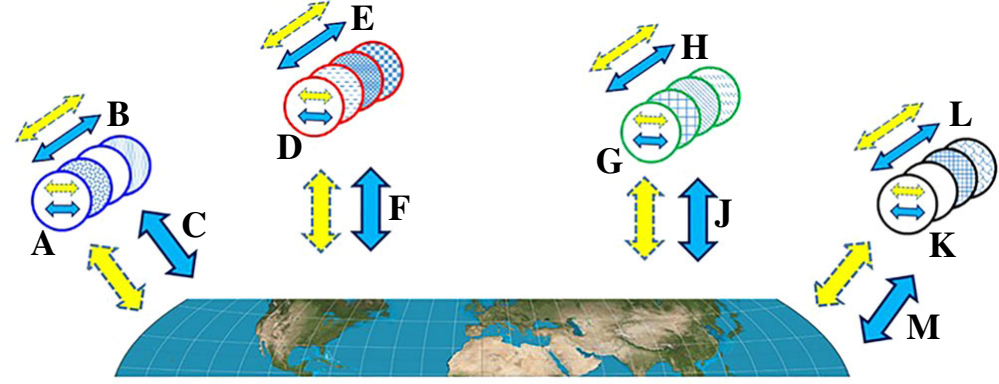
Species are defined by codes of core interactions. Organisms (shown as circles) are grouped in species (distinguished by blue, red, green and black borders), where each species is defined by the set of its core sustaining essential interactions (shown as blue arrows); these interactions include those that are internal to each organism in the species, between members of the species, and with other species and with the inanimate environment. Thus, the three sets of interactions in the species interaction code of each species (internal, intra-species and external) are respectively marked with {A, B, C}, {D, E, F}, {G, H, J} and {K, L, M}. Species also engage in circumstantial interactions (shown as yellow dashed arrows), which are not part of the species interaction code. Organisms within a species vary (shown as different fill patterns).

Figure 2 schematically summarizes the structure of the biosphere manifested through species codes of interactions.

In principle, each species could be characterized by a particular book of interactions. A detailed list of a species interactions for even the ‘simplest’ of species would challenge experts. We suggest, however, that a pairwise perspective of species might help clarify the concept of a species interaction code: given two related species, we might focus only on detectable interactions that distinguish the pair.

### (i) Voles and crows

Here are two examples of related species that can be distinguished by a few differences in their interaction codes.

**Voles.** The species termed prairie vole (*Microtus ochrogaster*) and meadow vole (*M. pennsylvanicus*) look very much alike, but the two species differ markedly in reproductive and social behaviours: prairie vole males are largely monogamous and social while meadow vole males are polygamous and solitary [25]. These interaction patterns are components of the codes that distinguish the two species. But meadow voles can be induced to express prairie vole interactions: Experimental insertion of a vasopressin receptor transgene into a specific site in the ventral forebrain of adult male meadow voles changes some of their reproductive and social behaviours to appear more similar to those of prairie voles; they become monogamous and friendly [26].

To date there is no information about the codes employed by female voles that distinguish the two different species. Presumably, the females of each species are attracted to interact with particular male behaviours. Other interaction code differences are likely to account for interactions with the different environments of these species.

**Crows.** Hooded crows (*Corvus cornix*) and carrion crows (*C. corone*) are very similar genetically (99.72% identical) to the point that they can produce fertile hybrid offspring [27]. The two species are in contiguity in large areas of Europe; why has not one species dominated and eliminated the other? What factor maintains two closely related species in the same physical environments? It turns out that the 0.28% genetic difference between the species includes the degree and pattern of feather pigmentation [28]; the two species, distinguished only by their appearance (carrions are totally black; hoodeds are partly grey), live in peace, defying Darwinian competition. It seems that crows prefer to mate with partners who look like their parents [29]. Thus, the two species can be distinguished by a single interaction code determinant of what Darwin has termed sexual selection [5]; what works works.

## 4. Reproduction and metabolism as essential interactions

Living systems manifest a great variety of interactions; however, the species interaction codes of all species include two essential properties: their ability to reproduce their kind and their ability to metabolize the energy and building blocks they require for maintenance and reproduction in their particular environment. Quite simply, species whose constituent organisms are not collectively capable of the interactions that metabolize and reproduce the species cannot survive [30].

Obviously not every organism within a species need metabolize and reproduce: organisms may exist in states of suspended animation (deep hibernation, dry seeds, spores) and only certain organisms may engage in reproduction. But metabolism and reproduction are interactions essential to the species as a collective whole, even though different species may carry out these essential interactions in different ways.

## 5. The role of species

Life, like matter, must adhere to the physical laws of nature [3]. Life, in its dependence on information and interaction, must accommodate the second law of thermodynamics, which dictates that information—ordered structure—will deteriorate spontaneously into disorder. One may argue that living systems are *open systems* and so may be able to resist the dictates of the second law; nevertheless, all multicellular organisms die. Boltzmann and (years later) Schroedinger have called attention to the paradox associated with the emergence of order and life [31, ch. 3], [32, ch. 6]. It is an observable fact that the persistence of life is accompanied by the re-production of its necessarily moribund organisms along with their metabolism.

How do reproduction and metabolism persist in a realm of universal individual death? Clearly, non-reproducing singletons do not last. Moreover, the loss of the singleton interrupts the networks in which the singleton acts. The institution of species provides one answer—species feature functionally replaceable singletons.

A single organism becomes a multiplicity as it reproduces. And multiplicity helps deal with entropy; the reproducing collective obeys the dictates of entropy, but the collective whole replaces organisms lost to the species by death.

From this perspective, we reason that the existence of multicellular life in a given environment requires functionally similar organisms in the aggregate framework of species. Individual organisms, by virtue of entropy, may come and go; only a collective species persists indefinitely in its environment—or at least until replaced by evolution.

The fossil and genetic records support the conclusion that multicellular life has appeared in the framework of evolving species for hundreds of millions of years; although one might possibly imagine other ways that the biosphere could have evolved to respond to the inevitable death of individual organisms, no equivalent to the species framework of multicellularity has yet been detected.

To paraphrase Darwin: we may say that multicellular life itself is the origin of species; if there be life, it must be in the form of a multitude of similar organisms organized as species. And, as we propose here, the members of a species are defined by their joint interaction code. From this viewpoint, a species interaction code is essential to multicellular life.

We have discussed species, interaction codes and the processes of encoding and decoding. We are now prepared to explore the possibility that evolution computes species using natural autoencoding. First, we shall briefly describe artificial autoencoding by computer, and then we shall apply the autoencoding concept to the natural autoencoding of species and evolution.

## 6. Artificial autoencoding

Autoencoding is a term associated with artificial intelligence, machine learning and artificial neural networks [33,34].

An artificial autoencoder is a computer program that extracts the defining features of the individuals in a given population, and then represents each individual as a set of values in a feature vector, or array. This *code* and its formative *encoding*, generated by artificial autoencoding, constitute a compact representation of the population and its individuals.

A typical artificial autoencoder is a neural network that, through an interactive training process, establishes encoding and decoding computations and the associated code. These computations can be used to encode each individual in a population, and to subsequently reconstruct each encoded individual from its respective code.

**Figure 3.**
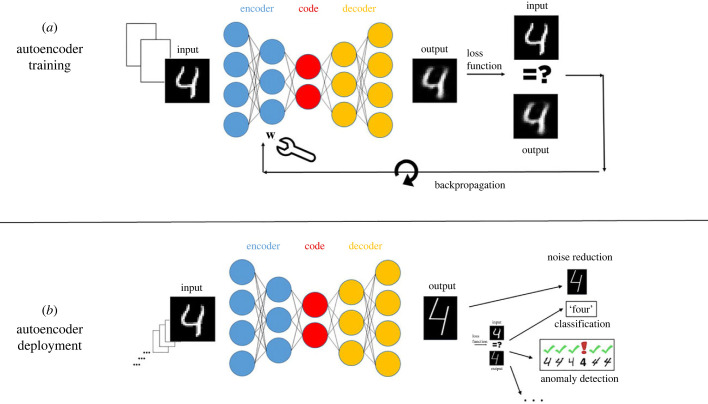
Artificial autoencoding. Typical autoencoders include three network-based elements: the encoder (blue circles), the code (red circles) and the decoder (orange circles). Individual inputs (handwritten digits, for example) are fed into the encoder, encoded as values in the code feature vector, and then reconstructed by the decoder. (*a*) During training, the differences between the output and the input are computed by a loss function, and, in an optimization process, the weights W of the connecting lines in the autoencoder’s neural net are adjusted to minimize the reconstruction loss. This is repeated using a finite set of examples. The process is termed backpropagation, and is often done using a gradient descent method. (*b*) Once training is completed, the autoencoder is deployed to perform its application task. Encoding and decoding are now done using the fixed code and edge weights to process an unbounded number of inputs from the domain of interest. The essence of artificial autoencoding is the creation and reshaping of the processes of encoders and decoders and thus of an entire working code system.

The machinery of an artificial autoencoder includes four elements: (i) *The encoder* receives input data regarding selected individuals, such as pixels of an image, audio signals, or measurements from some problem domain; the encoder outputs the learned feature vector with individual value assignments; this feature vector constitutes (ii) *The code*. (iii) *The decoder* accepts the code representing the encoding of a particular individual, and reconstructs the original input, such as the image or the sound segment. (iv) The fourth element is the training algorithm that builds the encoder and the decoder. See figure 3.

In the process that builds the encoder and decoder, the inputs and outputs are compared using a *loss function* to determine how close the reconstructed outputs are to the respective inputs. The internal parameters of the encoder and the decoder, which are commonly built using neural networks, are then adjusted and tuned, in an optimization process termed backpropagation, usually carried out by gradient descent, to minimize the loss function.

In artificial autoencoding, the training is unsupervised: the data are not labelled, so the autoencoder does not know what it is encoding. It is only required that the outputs be very similar to the corresponding inputs, specified by the loss function.

Once trained on a representative sample of a population, the autoencoder is able to encode and faithfully reconstruct many inputs from this population. Furthermore, certain autoencoders, termed variational, can use the code to generate new entities to be included in that population [35].

Artificial autoencoders enable many uses, including face recognition, image search, cleaning out image data by removing insignificant ‘noise’, anomaly detection, classification and more.

The array of features that comprises the numerical code may or may not include traits that a human observer would intuitively use to compactly describe the individual. Hence this code vector is often referred to as the *latent*, invisible vector, where only a properly trained decoder can ‘understand’ its features.

Consequently, artificial autoencoding can function without its human operators assigning meaning to the details of the process, that is, how the multitude of connection weights and nonlinear functions relate to the problem at hand. Autoencoding takes place in a ‘black box’, as it were, in which humans choose the network architecture and the activation functions, select the input, and develop the loss function. Autoencoder interpretability and explainability are still areas of research.

The opacity of autoencoding is important for our understanding of natural autoencoding of species and biosphere evolution, described below. Natural autoencoding takes place without any goals or processes selected by external agents.

## 7. Natural autoencoding

If codes define species, it would not be unreasonable to consider whether the formation and evolution of species might involve some form of autoencoding. *Natural autoencoding* would be an apt term if species evolution were to include the establishment of species code systems.

Let us analyse code, encoding and decoding in the contexts of life and evolution.

### (i) Natural codes

We have defined a code as a description of interactions, and not as actual interactions. What then constitutes the species code book? In what form might a biological code reside?

In our definition of *interactions*, we observed that sustained interactions are marked by *repetition and sequence* (§2). Accordingly, we propose that the molecular and behavioural features of the organism that enable the repetition of interactions constitute a description of such interactions. In other words, an infrastructure that anticipates a set of interactions is a code whose decoding materializes those interactions. A reusable biological network is a biological code ‘written’ for decoding.

We propose that the species code book is a composite of three forms of foundational information, both structural and dynamic: (1) the species germ-line genome, (2) the species physiology and (3) the arrangement of the species within a given ecosystem.

The germ-line genome is a diversity of DNA sequence information distributed within the population of organisms composing the species. But the germ-line genetic code alone cannot serve as the code book of the species; genes alone are not a readable record; genes have meaning only when expressed [14].

Danchin asserts that genetics alone is insufficient to account for biological function and that the underlying infrastructure of the whole cell, which he likens to a computer operating system, is essential [36].

The ways genes get expressed, are not encoded directly in DNA sequences: a single gene TNF-*α*, for example, gets expressed differently in embryonic development, inflammation, healing, immune system reactions, cancer, ageing, and other contexts [37].

TNF-*α* is not exceptional; most if not all proteins are pleiotropic—a single protein (derived from a single DNA sequence) will perform different functions in different contexts [1]. Moreover, different segments of a single gene sequence can be translated differently to express different proteins [14].

Another example of extra-genetic information is in cell division: the membrane of the daughter cell is built from the membrane of the dividing cell [38].

Consequently, the species code book must also include the molecular and physiological arrangements that describe the potential core interactions of the species—a description of *species physiology*. Consider bee species: the queen bee and the worker bees carry genes involved in enzymatic interactions, but there are no genes that directly encode honey. Honey, which is one of the outputs of interactions in the species interaction code of bees, is produced by worker bees in contexts in which particular enzymes and metabolic pathways get activated repeatedly in sequence.

Organisms also require ecological arrangements along with their genomes and physiology to survive. Bees, for example, need flowers, certain weather profiles and other information encoded within bee ecosystems. Thus the interaction with flowers which is part of the species interaction code of bees and of some flowers, is not directly coded in either the bees’ DNA or the flowers’ DNA, but is distributed.

Bees are only one example, the code books of all species include the genetics, physiology and ecology of the species.

### (ii) Natural encoding and decoding

The species infrastructure codebook is written by actual species interactions. Those interactions maintain the organisms of the species, strengthen the network infrastructure of the species and render it suitable for repetition—this constitutes the species interaction code.

The success of the input interactions confirms their potential to continue to maintain the organisms of the species in future rounds of interaction. In this way, input interactions are built into the species interaction code that forms a substrate for subsequent decoding.

Natural decoding is the continuing interactions of the organisms of the species in the species environment—genetic, physiological and ecological.

### (iii) From ecosystems to the biosphere

Every species that survives does so thanks to its integration within an ecosystem that provides the essential matter and metabolic energy on which the organisms of the species depend for survival.

Most plant species survive by exploiting sunshine, soil and water; insects may exploit plants and animals; animals may survive in predator–prey relationships; bacteria and fungi may exploit plants, animals, insects and other single-celled organisms.

But beyond relatively local ecosystems, species ecosystems are also integrated into larger ecosystems that include multiple species in complex relationships with other species; arrays of species link multiple levels of producers and predators and depend on energy and other resources passing between and within the various species.

The ultimate output of the process is the decoding of the global array of species and environments that together constitute the biosphere ecosystem. Thus the global output of species decoding is the maintenance and the evolution of the species and of the role of the species in the biosphere.

**Figure 4.**
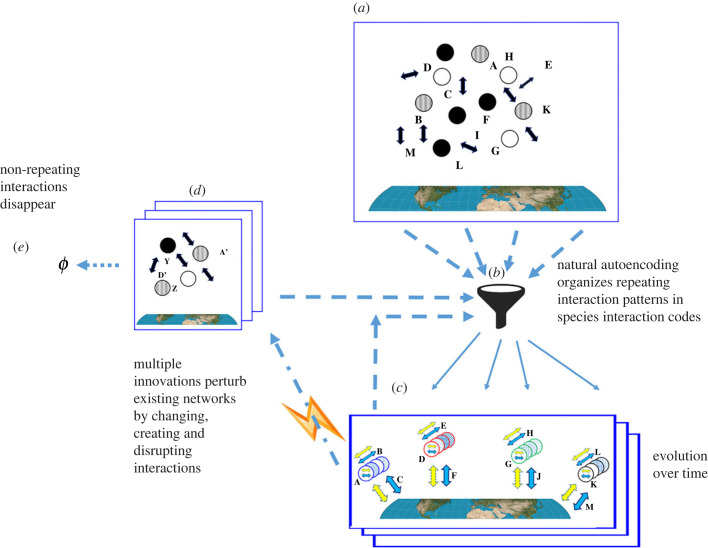
Evolution by natural autoencoding. (*a*) The plethora of interactions taking place in the biosphere are (*b*) funnelled into (*c*) networks of repeatable species interaction codes. Innovations (represented as a lightning bolt) can (*d*) perturb or destroy species interactions; two outcomes can take place: new fitted, repeatable interactions can arise and be funnelled into modified or new species; unfitted, non-repeating interactions disappear (*e*).

Figure 4 summarizes evolution by natural autoencoding.

### (iv) Integrated encoding and decoding

Conceptually, we treated natural encoding and decoding as separate processes. The interactions of living systems, however, are integrated into composite networks; it would be difficult to label a given reproductive, developmental or metabolic process as purely encoding or purely decoding.

For example, a sequence of DNA is decoded into a linear amino acid sequence, which itself is a coded description that encodes a functionally folded protein. This protein may then serve as an interaction code that is subsequently decoded into a structural protein, an enzyme or antibody that, in turn, can help encode additional metabolic, immune or social interactions that maintain and protect the species organisms and the ecosystem.

In [39], a machine learning process accounts for the encoding of the state of the body by the mammalian immune system.

**Figure 5.**
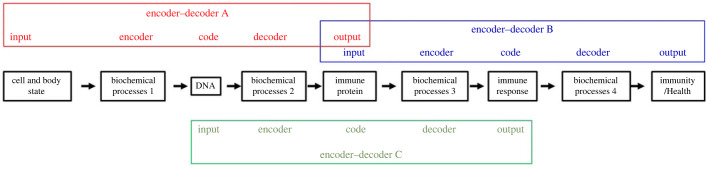
Linked assemblies of biosphere encoders and decoders. Multiple encoder–decoder pathways, including processes and structural entities, operate in parallel and are intertwined in a variety of ways forming chains and networks. Here for example, the immune system proteins are decoded from DNA by encoder–decoder A (shown in red above its constituent entities). Encoder–decoder B (blue) encodes the immune protein into an immune response pattern which is then decoded to generate health. Encoder–decoder B may be connected to additional entities and processes (not shown). Encoder–decoder C (shown in green below its constituent entities) can be seen as encoding a DNA sequence into a protein molecule code, which is then decoded into an immune response. More generally, every interaction, input, code or output of a given natural encoder–decoder pathway may also serve a function in another encoder–decoder pathway. The inputs to each encoding and decoding process may include additional entities beyond the ones shown here. Furthermore, each one of the many steps that constitute each encoder or decoder is an interaction in its own right, with its own description of input, encoder process, code, decoder process and output.

More generally, every interaction, input, code, or output of a given natural encoding–decoding process may also serve a function in another natural encoding–decoding process (figure 5). Every encoding or decoding process is itself an interaction (or set of interactions) associated with its own code.

Our concept of natural autoencoding is based on the realization that the biosphere is sustained by repeated interactions. Continuous encoding and decoding are central in maintaining and evolving this repetition. Encoding and decoding in the context of the biosphere, in contrast to the computer, refers to the indefinite repetition of biological interactions on planet earth in which one set of reactions—input information—is represented by a code that is decoded simultaneously into another set of reactions: output information.

### (v) Constraints

Interactions in general are organized by limitations, *constraints*, imposed on the interactors and on the environment; no stable structures can emerge when degrees of freedom are not limited [40]. Moreover, constraints decrease entropy by limiting the state-space of the system. Interactions result from constraints that channel the interactors to meet and interact. In fact, interactions themselves generate new constraints on what may follow. The cell membrane, for example, is formed by interactions between lipids, proteins and other molecules that effectively establish the boundary of the cell. This boundary keeps the contents of the cell together; localization is required for any interaction.

The force of gravity is a constraint that localizes land, water and atmosphere as media for life.

### (vi) Housekeeping autoencoding

We distinguish *housekeeping autoencoding* from *evolutionary autoencoding*.

Housekeeping autoencoding refers to existing species interactions that have not been perturbed by innovations that change species codes. Biological ‘business as usual’ is housekeeping—maintaining the house in a state of homeostasis.

Natural housekeeping autoencoding is similar to the error correction and noise reduction applications of artificial autoencoding in that the decoding process restores the integrity of the input. In artificial autoencoding noise reduction is computed in a trained autoencoder using the learned weights of the neural net (figure 3). In natural housekeeping autoencoding, the output is generated by subjecting the input to programs for DNA repair [41], cancer suppressor mechanisms [42], immune reactions, reproduction and programmed death [1], among others. These interaction programs are included in existing species interaction codes. Housekeeping autoencoding maintains the state of the individual by preserving and restoring a healthy, sustainable body state.

But preservation and restoration of individual homeostasis are not evolution. Evolutionary autoencoding refers to the evolution of new species interaction codes.

### (vii) Innovations and evolutionary autoencoding

Changes in species interaction codes emerge from innovations that lead to novel interactions in encoding and decoding. Innovations are perturbations that are not accommodated within the code of interactions of the existing species.

Perturbations and variations can take place both in the present code and in the environment—for example, genetically variant members of a hominoid species migrated out of Africa to evolve into the Neanderthal species in the European environment [43].

An innovation can enter the biosphere in a variety of ways and forms, be it a molecular mutation, an infecting pathogen, an invading species, a cancer cell, a change in nutrients or in solar radiation, a natural cataclysm, or a social or technological invention; witness, for example, the industrial revolution and global warming [44].

If an innovation is not integrated into a fitted configuration within networked species interaction codes, an unfitted interaction state can emerge; unfittedness can negatively affect molecules, cells, organisms, species and ecosystems; an innovation that does not integrate into a repeatable network will ultimately fail to survive and disappear (see figure 4).

## 8. Sexual reproduction, entropy and natural autoencoding

### (i) The challenge of sexual reproduction

Sexual reproduction has long presented a problem for the neo-Darwinian theory of evolution [45, ch. 3], [46, p. 265], [47]. Bell termed sexual reproduction the queen of problems in evolutionary biology [48, ch. 1].

To summarize the problem: natural selection teaches that evolution acts as an optimization process of individual fitness; yet, no matter how fit an individual may be, sexual reproduction, which involves random genetic recombination of parental genes, guarantees that one’s offspring will never inherit one’s exact genomic fitness. It seems counterproductive to select fit individuals and then to randomly disperse their genomes in the next generation. Despite many hypotheses, the problem is still open.

### (ii) Autoencoding and sexual reproduction

The natural autoencoding mechanism of evolution, in contrast to Darwinian natural selection, is not thwarted by the genetic randomization inherent in sexual reproduction; on the contrary, sexual reproduction, we reason, is essential in the natural autoencoding of most multicellular organisms.

As we wrote above, the survival of a species depends on the replacement of dead organisms by the reproduction of still living organisms (§4). The creation of a newborn results in a significant increase in order and complexity; this decrease in entropy is compensated by the increase in entropy generated by the unpredictable, random genomic recombination of the germ cells of the two parent organisms. Sexual reproduction fulfills a law of nature.

### (iii) Diversification, natural autoencoding and sex

The second law of thermodynamics dictates that organized structures will in time deteriorate and diversify. Diversification dismantles the optimum, the goal of natural selection. However, diversification is actually a necessary factor in natural autoencoding. An examination of artificial machine learning can help explain why.

Most forms of computer machine learning algorithms rely on inputs featuring randomly selected, diverse manifestations of the element to be learned: for example, artificial autoencoding of images of dogs begins by feeding the algorithm with many diverse representations of dogs [33]; teaching a computer to distinguish dogs from other entities (a classification task) may also require feeding diverse images of entities that are not dogs. A single dog photo, or even a million photos of a single dog will not suffice. The computer program needs to extract from many diverse photos of dogs and other entities the core features that characterize ‘dogness’. If the diversity of available input data is insufficient, some learning algorithms perturb or add random noise to the original data; this challenges the learning process to identify relevant features.

Natural autoencoding of a species interaction code, like computer machine learning, requires experience with randomly diverse examples of genomes and phenotypes borne by members of the species that, despite their genetic differences, thrive in the species environment. Sexual reproduction enables the continuous input into the species environment of organisms bearing workable arrays of genomic diversity.

Moreover, random diversification by sexual reproduction defines the functional extent of genome variation operating in the species. Failure of sexual interactions to generate reproducing offspring limits the effects of the second law of thermodynamics which guarantees that random genetic mutations will occur. Sexual reproduction culls the species of ‘bad’ genes and gene combinations; genomes of sexually reproducing organisms that fail to be propagated into the next generation are weeded out in the process of reproduction.

Sexual reproduction not only establishes the functional diversity of a species’s DNA genome; the sex act also tests physiological, social and ecological codes within the species: Attraction, courting, nesting, and rituals, physical and symbolic, mark the sexual reproduction of many species. Sex establishes, maintains and tests many interaction codes in the given species’s environment.

Sexual reproduction may be a problem for evolutionary concepts based on individual optimization, but not for a concept of evolution based on natural autoencoding of ‘what works’. Sexual reproduction removes species interactions that do not work in the species environment and it does so by design, and not by unpredictable accident. Sexual reproduction thus enables the species as a whole to autoencode itself genetically, physiologically and ecologically.

We propose that sexual reproduction is essentially universal in multicellular species because the repetition of living interactions must continue despite the inevitable diversification dictated by the second law of thermodynamics. Sex, from this perspective is foundational, not paradoxical.

As we mentioned in §3, the concept of bacterial species is controversial [22]. Moreover, bacteria do not engage in sexual reproduction; however, it has been suggested that horizontal gene transfer may play a role in defining the borders of species in bacteria [49], along with compensatory genomic diversification and increased entropy.

### (iv) Summary of sexual reproduction, entropy and natural autoencoding

**Figure 6.**
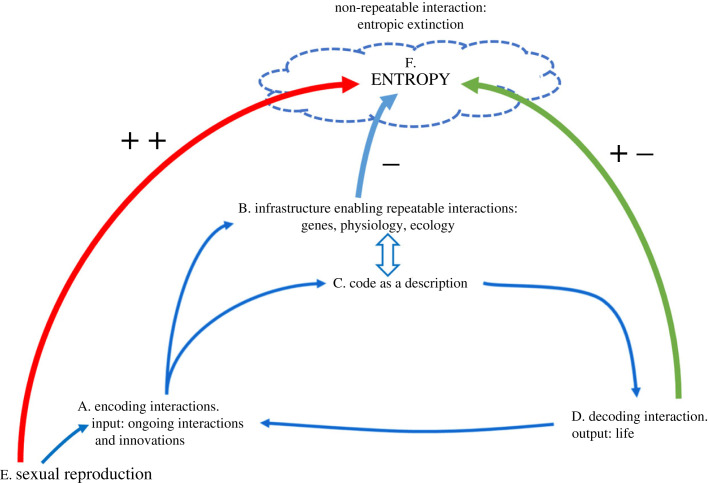
Schematic view of natural autoencoding, sexual reproduction and the relevant effects on entropy. See text for details.

Figure 6 summarizes the links between natural autoencoding, sexual reproduction and entropy. The mechanism of natural autoencoding is outlined in the figure in the items marked A, B, C and D; the role of sexual reproduction is labelled E, and entropy is marked F; the arrows designate influences and relationships. Encoding interactions, marked A in the figure, are composed of both ongoing interactions and innovations, such as molecular mutations, new invading parasites or physical perturbations and environmental changes.

This input proceeds in two separate pathways: A to B and A to C. Pathway A to B marks those repeatable, surviving genetic, physiological and ecological interactions that compose the network infrastructure that enables the repetition of interactions; this infrastructure, constitutes a description of species interaction codes (shown as a wide bidirectional arrow connecting B and C). Innovations that integrate into supporting networks may change species interaction codes and thus generate new species. Encoding interactions not only generate the species interaction codes, input interactions also activate existing infrastructure networks, shown as the connecting arrows from A to C and C to D. This decoding of the species interaction code infrastructure realizes the outputs that constitute life. The interactions of life (D) feed back into the input (A) that generates and activates the species interaction codes (C) of the living, evolving biosphere (A–D).

The organized biosphere (A–D), like all of material existence, constantly generates a degree of compensatory disorganization or lost energy.

The enabling structure of life and its natural autoencoding (A–D) reduces, entropy; this is shown as the thick blue arrow from B to F, labelled with a minus sign (−). sexual reproduction (E), which is an input into A, compensates life’s overall decrease of entropy by enhancing the entropy of organismal replacement (F) through random genomic diversification, marked by the thick red arrow (++).

The interactions of life that organize the biosphere also contribute an increase in entropy by lost heat, degradation, illness, mortality, individual differences and destruction of organization (thick green arrow marked + −). Non-repeatable interactions are lost to entropic extinction.

## 9. Comparing artificial and natural autoencoding

**Figure 7.**
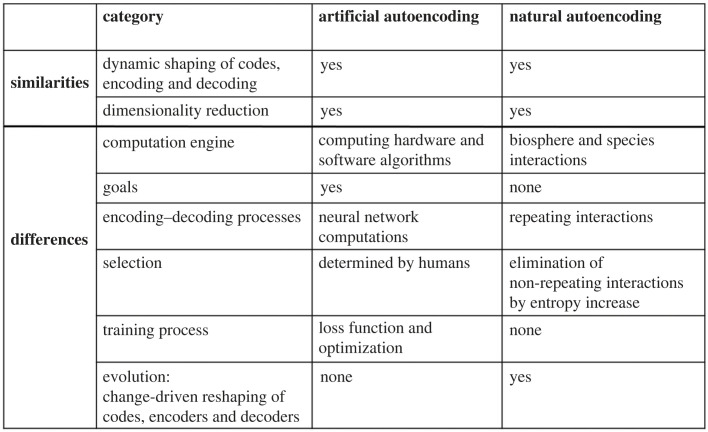
Summary comparison of natural and artificial autoencoding. See text for details.

Figure 7 summarizes the similarities and differences between artificial and natural autoencoding. Both processes shape code systems and both reduce the dimensionality of input data to essentials. However, natural autoencoding aims at no designated goals and has no training process involving loss functions and optimizations. Natural autoencoding does not use computing hardware or software, but is the outcome of biosphere and species interactions in which repeating interactions become species interaction codes; non-repeating interactions are eliminated selectively by entropy. Moreover, natural autoencoding, in response to innovations, spontaneously evolves its encoding, codes or decoding processes and interactions.

Evolution seems to have experienced natural autoencoding millions of years before humans developed artificial autoencoding. We arrived at the concept of natural autoencoding by way of artificial computer autoencoding. However, from the perspective of evolutionary time, artificial autoencoding, is a ‘non-conventional’ variant of the natural process that preceded it.

## 10. On evolution as a machine learning process

Recently, Vanchurin and colleagues have applied machine learning concepts and thermodynamic principles to develop a theory of evolution as multilevel learning [50,51].

The main differences between the ideas of natural autoencoding and the theory of Vanchurin and colleagues are as follows. Vanchurin and colleagues state that the biosphere learns to compute a fitness function, uses it to compute the loss function and then optimizes this loss function. For example, 'We make the case that loss function, which is central to the learning theory, can be usefully and generally employed as the equivalent of the fitness function in the context of evolution' [50]. By contrast, the evolutionary mechanism of autoencoding proposed here does not compute fitness, and does not use a loss function. Furthermore, in the computations carried out by natural autoencoding in response to innovations there is no optimization: ‘what works works’. Natural autoencoding is based only on the observed preservation of repeating interactions.

## 11. Humans and the biosphere

The rapid expansion of the human population in the past ten thousand years owes its onset to the domestication of species of plants and animals by humans; humans chose to propagate only those species innovations that satisfied perceived human needs. Whether or not one accepts Darwin’s idea of Natural Selection as a ‘law of nature’ [52–54], the ‘natural right’ of domination by the ‘fittest’ has influenced many aspects of human culture including ethics, economics, governance, racial relations, social organization and education [55–59]. The centrality of domination in natural selection is problematic both for our understanding of the biosphere and for our behaviour within it.

Thomas Kuhn has pointed out the blinding power of entrenched paradigms in science [60]. Traditional studies of evolution assume survival of the fittest as a given, even when they attempt to account for group cooperation [6].

Misunderstanding alone is tolerable; misguided action is not. The spirit of domination underlies much of the irresponsible human behaviour that is now changing the biosphere.

Natural selection is not sufficiently sensitive to the world wide web of cooperative interactions among species and environments required to maintain a biosphere friendly to the well-being of the human species. We hope that an appreciation of natural autoencoding and survival of the fitted will help support the movement to change the human interactions presently damaging the biosphere.

## 12. Modelling and simulation

Here, we have introduced the concept of natural autoencoding; however reasonable, concepts alone do not suffice. Further work needs to be done to support or refute the idea. We are now developing architectures and algorithms for computer modelling and mathematical definitions of natural autoencoding. We use standard and new artificial autoencoding techniques, including neural networks, agent-based modelling, principal component analysis algorithms, and property-preserving mathematical transformations of large data structures. We do not claim that the forces and interactions of nature can be mapped directly to elements of any specific modelling technique. Nevertheless, these models can extend our understanding of the biosphere and might even provide new tools in computer science.

## Data Availability

This article has no additional data.

## References

[1] Cohen IR. 2000 Tending Adam’s garden: evolving the cognitive immune self. London, UK: Elsevier.

[2] Cohen IR. 2016 Updating Darwin: information and entropy drive the evolution of life. F1000Research **5**, 2808. (10.12688/f1000research.10289.1) 28105315 PMC5200945

[3] Cohen IR, Marron A. 2020 The evolution of universal adaptations of life is driven by universal properties of matter: energy, entropy, and interaction. F1000Research **9**, 626. (10.12688/f1000research.24447.3) 32802320 PMC7416572

[4] Koonin EV. 2011 The logic of chance: the nature and origin of biological evolution. Upper Saddle River, NJ: FT Press.

[5] Darwin C. 1860 On the origin of species by means of natural selection, or the preservation of favoured races in the struggle for life. London, UK: John Murray. PMC5184128 30164232

[6] Nowak MA. 2006 Five rules for the evolution of cooperation. Science **314**, 1560-1563. (10.1126/science.1133755) 17158317 PMC3279745

[7] Sachs JL, Mueller UG, Wilcox TP, Bull JJ. 2004 The evolution of cooperation. Q. Rev. Biol. **79**, 135-160. (10.1086/383541) 15232949

[8] Blaser MJ. 2014 The microbiome revolution. J. Clin. Invest. **124**, 4162-4165. (10.1172/JCI78366) 25271724 PMC4191014

[9] Simard SW. 2018 Mycorrhizal networks facilitate tree communication, learning, and memory. In Memory and learning in plants (eds F Baluska, M Gagliano, G Witzany), pp. 191-213. Berlin, Germany: Springer.

[10] Rosenberg E, Koren O, Reshef L, Efrony R, Zilber-Rosenberg I. 2007 The role of microorganisms in coral health, disease and evolution. Nat. Rev. Microbiol. **5**, 355-362. (10.1038/nrmicro1635) 17384666

[11] Judge A, Dodd M. 2020 Metabolism. Essays Biochem. **64**, 607-647. (10.1042/EBC20190041) 32830223 PMC7545035

[12] Cohen IR. 2006 Informational landscapes in art, science, and evolution. Bull. Math. Biol. **68**, 1213-1229. (10.1007/s11538-006-9118-4) 16832743 PMC7088857

[13] Shannon CE. 1948 A mathematical theory of communication. Bell Syst. Tech. J. **27**, 379-423. (10.1002/j.1538-7305.1948.tb01338.x)

[14] Cohen IR, Atlan H, Efroni S. 2016 Genetics as explanation: limits to the human genome project. Encyclopedia of Life Sciences. Chichester, UK: John Wiley & Sons.

[15] Doige CA, Day T. 2012 A typology of undergraduate textbook definitions of heat across science disciplines. Int. J. Sci. Educ. **34**, 677-700. (10.1080/09500693.2011.644820)

[16] Gleick J. 1993 Genius: the life and science of Richard Feynman. New York, NY: Vintage.

[17] Rovelli C. 2017 Carlo Rovelli—all reality is interaction. See https://www.wnyc.org/story/59a21fbf4616dd86cb8fc341 (accessed January 2023).

[18] Igamberdiev AU. 2018 Time and life in the relational universe: prolegomena to an integral paradigm of natural philosophy. Philosophies **3**, 30. (10.3390/philosophies3040030)

[19] Le Poidevin R, Peter S, Andrew M, Cameron RP. 2009 The Routledge companion to metaphysics. Abingdon, UK: Routledge.

[20] Oxford University Press. 2022. Code [definition]. See https://www.oed.com/.

[21] Mallet J. 1995 A species definition for the modern synthesis. Trends Ecol. Evol. **10**, 294-299. (10.1016/0169-5347(95)90031-4) 21237047

[22] Chun J et al. 2018 Proposed minimal standards for the use of genome data for the taxonomy of prokaryotes. Int. J. Syst. Evol. Microbiol. **68**, 461-466. (10.1099/ijsem.0.002516) 29292687

[23] Barbieri M. 2012 Codepoiesis—the deep logic of life. Biosemiotics **5**, 297-299. (10.1007/s12304-012-9162-4)

[24] Barbieri M 2015 Code biology: a new science of life. Cham, Switzerland: Springer.

[25] Gruder-Adams S, Getz LL. 1985 Comparison of the mating system and paternal behavior in *microtus ochrogaster* and *M. pennsylvanicus*. J. Mammal. **66**, 165-167. (10.2307/1380976)

[26] Lim MM, Wang Z, Olazábal DE, Ren X, Terwilliger EF, Young LJ. 2004 Enhanced partner preference in a promiscuous species by manipulating the expression of a single gene. Nature **429**, 754-757. (10.1038/nature02539) 15201909

[27] Wolf JB, Bayer T, Haubold B, Schilhabel M, Rosenstiel P, Tautz D. 2010 Nucleotide divergence vs. gene expression differentiation: comparative transcriptome sequencing in natural isolates from the carrion crow and its hybrid zone with the hooded crow. Mol. Ecol. **19**, 162-175. (10.1111/j.1365-294X.2009.04471.x) 20331778

[28] Poelstra JW et al. 2014 The genomic landscape underlying phenotypic integrity in the face of gene flow in crows. Science **344**, 1410-1414. (10.1126/science.1253226) 24948738

[29] Metzler D, Knief U, Peñalba JV, Wolf JB. 2020 Assortative mate choice and epistatic mating-trait architecture induces complex hybrid-zone movement. *BioRxiv*. 10.1111/evo.14386 34694633

[30] Dupré J, O’Malley MA. 2013 Varieties of living things: life at the intersection of lineage and metabolism. In Vitalism and the scientific image in post-enlightenment life science, 1800–2010 (eds S Normandin, CT Wolfe), pp. 311-343. Berlin, Germany: Springer.

[31] Boltzmann L. 2012 The second law of thermodynamics. In Theoretical physics and philosophical problems: selected writings (ed. B McGuinness). Dordrecht, The Netherlands: D Reidel Publishing Company.

[32] Schrödinger E. 1944 What is life? The physical aspect of the living cell and mind. Cambridge, UK: Cambridge University Press.

[33] Goodfellow I, Bengio Y, Courville A. 2016 Deep learning. Cambridge, MA: MIT Press. See http://www.deeplearningbook.org.

[34] Kramer MA. 1991 Nonlinear principal component analysis using autoassociative neural networks. AlChE J. **37**, 233-243. (10.1002/aic.690370209)

[35] Kingma DP, Welling M. 2013 Auto-encoding variational bayes. Preprint. (https://arxiv.org/abs/1312.6114)

[36] Borriss R, Danchin A, Harwood CR, Médigue C, Rocha EP, Sekowska A, Vallenet D. 2018 Bacillus subtilis, the model gram-positive bacterium: 20 years of annotation refinement. Microb. Biotechnol. **11**, 3-17. (10.1111/1751-7915.13043) 29280348 PMC5743806

[37] Romanowska-Próchnicka K, Felis-Giemza A, Olesińska M, Wojdasiewicz P, Paradowska-Gorycka A, Szukiewicz D. 2021 The role of TNF-*α* and anti-TNF-*α* agents during preconception, pregnancy, and breastfeeding. Int. J. Mol. Sci. **22**, 2922. (10.3390/ijms22062922) 33805757 PMC7998738

[38] Mazia D. 1961 The cell, pp. 77-412. London, UK: Elsevier.

[39] Cohen IR, Efroni S. 2019 The immune system computes the state of the body: crowd wisdom, machine learning, and immune cell reference repertoires help manage inflammation. Front. Immunol. **10**, 10. (10.3389/fimmu.2019.00010) 30723470 PMC6349705

[40] Grotzinger JP, Bowring SA, Saylor BZ, Kaufman AJ. 1995 Biostratigraphic and geochronologic constraints on early animal evolution. Science **270**, 598-604. (10.1126/science.270.5236.598)

[41] Sancar A, Lindsey-Boltz LA, Ünsal-Kaçmaz K, Linn S. 2004 Molecular mechanisms of mammalian DNA repair and the DNA damage checkpoints. Annu. Rev. Biochem. **73**, 39-85. (10.1146/annurev.biochem.73.011303.073723) 15189136

[42] Soussi T. 2000 The p53 tumor suppressor gene: from molecular biology to clinical investigation. Ann. NY Acad. Sci. **910**, 121-139. (10.1111/j.1749-6632.2000.tb06705.x) 10911910

[43] Mellars P. 2004 Neanderthals and the modern human colonization of Europe. Nature **432**, 461-465. (10.1038/nature03103) 15565144

[44] Rosenzweig C et al. 2008 Attributing physical and biological impacts to anthropogenic climate change. Nature **453**, 353-357. (10.1038/nature06937) 18480817

[45] Dawkins R. 1976 The selfish gene. Oxford, UK: Oxford University Press.

[46] Ridley M. 1993 Evolution. Oxford, UK: Blackwell Scientific.

[47] Livnat A, Papadimitriou CH. 2016 Sex as an algorithm: the theory of evolution under the lens of computation. Commun. ACM **59**, 84-93. (10.1145/2934662)

[48] Bell G. 2019 The masterpiece of nature: the evolution and genetics of sexuality. Abingdon, UK: Routledge. Originally published in 1982.

[49] De la Cruz F, Davies J. 2000 Horizontal gene transfer and the origin of species: lessons from bacteria. Trends Microbiol. **8**, 128-133. (10.1016/S0966-842X(00)01703-0) 10707066

[50] Vanchurin V, Wolf YI, Katsnelson MI, Koonin EV. 2022 Toward a theory of evolution as multilevel learning. Proc. Natl Acad. Sci. USA **119**, e2120037119. (10.1073/pnas.2120037119) 35121666 PMC8833143

[51] Vanchurin V, Wolf YI, Koonin EV, Katsnelson MI. 2022 Thermodynamics of evolution and the origin of life. Proc. Natl Acad. Sci. USA **119**, e2120042119. (10.1073/pnas.2120042119) 35131858 PMC8833196

[52] Bradley B. 2022 Natural selection according to Darwin: cause or effect? Hist. Phil. Life Sci. **44**, 1-26. (10.1007/s40656-022-00485-z) PMC9001397 35411477

[53] Byerly HC. 1983 Natural selection as a law: principles and processes. Am. Nat. **121**, 739-745. (10.1086/284099)

[54] Reed ES. 1981 The lawfulness of natural selection. Am. Nat. **118**, 61-71. (10.1086/283801)

[55] Auerswald PE, Branscomb LM. 2003 Valleys of death and Darwinian seas: financing the invention to innovation transition in the united states. J. Technol. Transf. **28**, 227-239. (10.1023/A:1024980525678)

[56] Bergman J. 2014 The Darwin effect: it’s influence on nazism, eugenics, racism, communism, capitalism & sexism. Green Forest, AR: New Leaf Publishing Group.

[57] Browning L, Thompson K, Dawson D. 2017 From early career researcher to research leader: survival of the fittest? J. Higher Educ. Policy Manage. **39**, 361-377. (10.1080/1360080X.2017.1330814)

[58] Mayr E. 2000 Darwin’s influence on modern thought. Sci. Am. **283**, 78-83. (10.1038/scientificamerican0700-78) 10881312

[59] Wyllie IG. 1959 Social Darwinism and the businessman. Proc. Am. Phil. Soc. **103**, 629-635.

[60] Kuhn TS. 1970 The structure of scientific revolutions, vol. 111. Chicago, IL: University of Chicago Press.

